# Identification performance of MALDI-ToF-MS upon mono- and bi-microbial cultures is cell number and culture proportion dependent

**DOI:** 10.1007/s00216-019-02080-x

**Published:** 2019-09-05

**Authors:** Christoph Mörtelmaier, Suchita Panda, Iain Robertson, Mareike Krell, Marilena Christodoulou, Nicole Reichardt, Imke Mulder

**Affiliations:** 4D Pharma Research Ltd., Cornhill Road, Aberdeen, AB25 2ZS UK

**Keywords:** MALDI-ToF, Biotyping, Viable cell count, Mixed culture

## Abstract

Biotyping using matrix-assisted laser desorption ionization-time of flight (MALDI-ToF) mass spectroscopy (MS) has revolutionized microbiology by allowing clinicians and scientists to rapidly identify microbes at genus and species levels. The present study extensively assesses the suitability and reliability of MALDI-ToF biotyping of 14 different aerobic and anaerobic bacterial species as pure and mixed cultures. Reliable identification at species level was possible from biomaterial of older colonies and even frozen biomaterial, although this was species dependent. Using standard instrument settings and direct application of biomaterial onto the MALDI-ToF target plates, it was determined that the cell densities necessary for completely reliable identification of pure cultures varied between 2.40 × 10^8^ and 1.10 × 10^10^ viable cell counts (VCCs) per mL, depending on the species. Evaluation of the mixed culture algorithm of the Bruker Biotyper^®^ software showed that the performance of the algorithm depends greatly on the targeted species, on their phylogenetic distance, and on their ratio of VCC per mL in the mixed culture. Hence, the use of MALDI-ToF-MS with incorporation of the mixed culture algorithm of the software is a useful pre-screening tool for early identification of contaminants, but due to the great variability in performance between different species and the usually unknown percentage of the possible contaminant in the mixture, it is advisable to combine this method with other microbiology methods.

**Electronic supplementary material:**

The online version of this article (10.1007/s00216-019-02080-x) contains supplementary material, which is available to authorized users.

## Introduction

The use of matrix-assisted laser desorption ionization-time-of-flight mass spectrometry (MALDI-ToF-MS) has made the identification of microorganisms quick, reliable, and cost-effective and hence allowed high-throughput identification (so called “biotyping”) [1]. This has revolutionized the field of clinical microbiology, but it is also gaining traction in research [2]. The characterization of mono-microbial samples yields highly reliable identification for around 90% of tested samples [1, 3–5]. The successful identification of bacterial samples with MALDI-ToF-MS is dependent on the availability and quality of reference spectrum libraries. Additionally, reference spectra may not give optimal identification results, because different growth conditions or intra-species variability between test organisms and reference organisms is reflected in spectrum variations [6]. The creation of in-house reference libraries is a way to overcome these limitations and moreover, to improve identification specificity and efficiency [7].

In many applications, only low bacterial biomass may be available and different approaches have been used to determine the minimum concentration of cell material needed for identification of bacteria using MALDI-ToF-MS. Many of these studies focus on direct identification of bacteria in clinical samples, e.g., from urinary or respiratory tract infections [8–11]. In these arenas, species differences in detection limits were reported when different MALDI-ToF systems were used, such as the Bruker Autoflex, the Bruker Microflex, the Bruker Ultraflex MALDI-ToF-MS, or the bioMérieux VITEK^®^ MS MALDI-TOF. For example, Ferreira and colleagues [12] showed *Escherichia coli* could be identified at species level at 8 × 10^4^ viable cell count (VCC) per mL using a Bruker Autoflex, whereas *Enterococcus faecalis* did not reach consistently high identification scores even at 5 × 10^5^ VCC per mL. Other studies showed that the detection limit could be improved by applying optimized protocols (including diafiltration and specific extraction methods) and improved algorithms for spectral analyses [13–15].

Bacterial species commonly exist in polymicrobial communities; however, pure bacterial cultures are necessary for diagnostics purposes as well as for most uses in research and biotechnology. Traditional methods to assess the purity of bacterial cultures involve streaking of purity plates with often long incubation times and subsequent assessment (morphology, characteristics) and identification (16S rRNA gene sequencing, MALDI-ToF-MS). As these methods are relatively time consuming, researchers have attempted to establish ways to identify biological samples containing two or more different species by MALDI-ToF-MS. In an early approach, an algorithm was developed, which was able to identify mixed cultures of two bacteria present in a reference library [16]. In the work of Mahe and colleagues [17], a broad set of mixed samples was tested, which contained two different species in different volumetric ratios using bioMérieux’s VITEK^®^ system. In more than 60% of the measured spectra across all applied mixtures, both organisms could be identified at least at the genus level, and a link between taxonomic distance and measured raw spectra of mixed cultures was shown. In the spectrum of a polymicrobial sample, peaks can be assigned to the single species in the mixture to great extent, which allows identifying the single species inside the mixture using a biomarker-based approach for up to six species with the same optical density at 600 nm (OD_600_) inside a mixture [18]. A recent study used samples from blood cultures to perform an evaluation of the Bruker Biotyper^®^ mixed culture algorithm, which can identify bi-microbial mixtures. The data highlighted the great variability between combinations of different species and their mixture ratios, with some species more easily identified in polymicrobial samples than others [19]. Similarly, mixed cultures of *Escherichia coli* and *Enterococcus faecium*, *E*. *coli* and *Pseudomonas aeruginosa*, and *E*. *coli* with *Enterococcus faecalis* from BacT/ALERT blood culture bottles measured on a Bruker Microflex were identified as *E*. *coli* pure cultures [9, 20]. Furthermore, in routine clinical diagnostics, identification of polymicrobial samples was not always successful, for example, only in 10% [21] or 30% [10] of polymicrobial samples of blood culture pellets could both species be identified. In a most recent approach, a synthetic mixture spectrum was combined with a statistical assessment by a jackknife model to achieve 80% sensitivity for bi-microbial samples [22]. A recent detailed review on this topic covering many different combinations of species and different approaches was published in 2018 [23].

The present study focused on the identification of bacterial colonies and liquid cultures across a broad phylogenetic range using the Bruker Biotyper^®^ MALDI-ToF-MS. The application of VCC and the high number of measurements required made it necessary to investigate a way to preserve the bacterial biomass long enough. Therefore, the effect of sample biomass maturity on the reliability and quality of MALDI-ToF identification was investigated during long-term incubation and revealed the necessity to freeze the cell pellets. We then evaluated the required minimum concentration of VCC per mL for a reliable identification of bacteria in liquid pure cultures by using a linear model. There is always a risk of contamination in the production of bacterial biomass from liquid cultures, and ensuring the purity of a bacterial culture with purity plates and subsequent colony analyses is a time-consuming process. The direct analysis of a culture or a cell pellet originating from the bacterial suspension would be a much quicker method. We therefore assessed the detection of bacteria in mixtures containing different species of various ratios.

## Material and methods

Figures were made using GraphPad 8.1.0 (325). Statistical calculations were done using GraphPad 8.1.0 (325) and Microsoft Excel for Office 365 MSO 16.0.11601.20174.

### Instrument settings

All samples were measured on a Bruker Microflex Lt^®^ MALDI-ToF mass spectrometer (Bruker Daltonics, Germany) using the method “MBT_AutoX.” The laser was set at a shot rate of 60 Hz with the measuring raster (spiral_small). Spectra were accumulated in the MS/parent mode (240 shots). Peaks were evaluated with the processing method “MBT_Process” using flexControl (version 3.4, Bruker Daltonics, Germany), peak detection algorithm “centroid” (signal-to-noise threshold of 2, a minimum intensity threshold of 600 a.u. and a maximum number of peaks of 300, a peak width of 4 *m*/*z* and height of 90%, baseline subtraction with the method “TopHat” for a peak resolution > 400). Spectra which did not meet the minimum quality requirements gave a flatline spectrum as output. The instrument was calibrated by using Bacterial Test Standard (BTS) from Bruker. All spectra were compared with reference spectra of the BDAL database (version 7.0.0.0) and with main spectrum profiles (MSPs) created. Each spot was overlaid with 1 μL of HCCA (10 mg/mL α-cyano-4-hydroxybenzoic acid (HCCA)) in 50% acetonitrile, 47.5% water, and 2.5% trifluoroacetic acid (TFA) matrix (Bruker Daltonics, Germany) before measurement.

### Bacterial strains and culture conditions

Fourteen bacterial strains were selected to include a variety of Gram-positive, Gram-negative, aerobic, and anaerobic bacterial species. Strain origin and properties are summarized in Table 1. Bacteria were streaked from glycerol stocks (stored at − 80 °C) onto YCFA agar [24] (E&O Laboratories, UK) and grown for 3 days. All strains were grown anaerobically at 37 °C apart from *Bacillus subtilis* (grown aerobically at 30 °C), and *Pseudomonas aeruginosa* and *Staphylococcus aureus* (both grown aerobically at 37 °C).

**Table 1:** Overview of bacterial species used in this study. The source of the microorganism was either the culture collection of 4D pharma plc (fecal isolates from healthy human donors) or public culture collections: American Type Culture Collection (ATCC), National Collection of Type Cultures (NCTC), National Collection of Industrial, Food and Marine Bacteria (NCIMB). The number of Main Spectra Profiles (MSP) gives the number of database entries for a species in the database of Bruker Daltonics (BDAL) and in the custom in-house database (in house). All bacteria were used in the incubation time experiment (ITE); nine species were selected for the determination of an ideal cell concentration for ID of pure cultures as well as ID performance in mixed cultures (MC). [fac. anaerobic: facultative anaerobic]

Microorganism	Source/strain		Gram nature	MSP (BDAL/in-house)	Experiments used in
*Bacillus subtilis*	NCTC 10400	Aerobic	+	14/0	ITE
*Blautia stercoris*	4D pharma plc	Anaerobic	+	0/1	ITE
*Bifidobacterium breve*	4D pharma plc	Anaerobic	+	5/0	ITE, MC
*Bifidobacterium longum*	4D pharma plc	Anaerobic	+	6/0	ITE
*Clostridium sporogenes*	ATCC 3584	Anaerobic	+	7/0	ITE, MC
*Enterococcus faecalis*	4D pharma plc	Fac. anaerobic	+	11/0	ITE, MC
*Enterococcus gallinarum*	4D pharma plc	Fac. anaerobic	+	3/1	ITE, MC
*Escherichia coli*	ATCC 11750	Fac. anaerobic	−	14/0	ITE, MC
*Megasphaera massiliensis*	4D pharma plc	Anaerobic	−	0/3	ITE, MC
*Parabacteroides distasonis*	4D pharma plc	Anaerobic	−	9/0	ITE, MC
*Pseudomonas aeruginosa*	NCTC 12924	Aerobic	−	9/0	ITE
*Roseburia hominis*	4D pharma plc	Anaerobic	Variable	0/1	ITE
*Salmonella enterica* serovar *Typhimurium*	NCIMB 10248	Fac. anaerobic	−	18/0	ITE, MC
*Staphylococcus aureus*	NCIMB 9518	Aerobic	+	14/0	ITE, MC

### Identification of bacterial colonies over longer incubation time

A single colony of each bacterium was inoculated into individual culture tubes containing 10 mL YCFA broth and incubated for 24 h before streaking again on YCFA agar in triplicate. After 24 h, three single colonies from each agar plate (2 spots per colony, 6 spots for each strain in total) were identified using the MALDI-ToF (Biotyper^®^ 4.0.19 Bruker) as per the user manual provided by Bruker using the default parameter settings. Colonies of each strain were subsequently measured in the same way every 24 h for up to 21 days. Results were classified following the standard identification scores provided by the Bruker Biotyper^®^ software: ID score < 1.7 indicates “not reliable ID,” “Genus level ID” is a reliable identification and probable genus level ID with an ID score > 1.7, and ID score > 2.0 indicates a reliable identification secure Genus level ID (probable Species level). If the measurement resulted in a flatline spectrum or the tested organism was not listed in the 10 best score results, a value of 1 was used. The maximum scores for each species per day were used. *Salmonella Typhimurium* is a serovar of the species *Salmonella enterica*, and therefore, all serovar main spectrum profile (MSP) entries of *S*. *enterica* were considered for the species identification [25]. For the species *Roseburia hominis*, *Blautia stercoris*, and *Megasphaera massiliensis*, MSPs were created in-house according to recommendations of the manufacturer as these species had no entries in the BDAL database (Table 1). For *Enterococcus gallinarum*, an additional MSP was created, although reference entries were present. The scores of the created MSPs and the MSPs provided by BDAL database were compared to illustrate the increase of the ID score when a custom MSP exactly matched the strain and culture conditions. For all other identifications of *E*. *gallinarum* in the following chapters, the created MSP was used in addition with the MSPs in the BDAL database.

### Determination of minimum concentration for reliable identification of strains using MALDI-ToF

Nine strains (Table 1) were selected for determining their minimum concentration for reliable identification (limit of detection), including four Gram-negative species and five Gram-positive species and also including species belonging to the same genus (*Enterococcus gallinarum* and *Enterococcus faecalis*). Bacterial growth curves were performed in duplicate in 10 mL YCFA broth using a 1% inoculum from a 24 h pre-culture. Optical density at 600 nm (OD_600_) was measured every 2 up to 16 h. The absorbance readings were plotted against time to obtain a sigmoid curve from which the different growth stages were determined (lag phase, logarithmic phase, stationary phase). Bacterial cultures were then grown again (20 mL, 1% inoculum from 24 h pre-culture) in triplicate. VCCs were determined; cell material was harvested at logarithmic or late logarithmic growth phase (1-mL aliquots, 12,000×*g*, 3 min) to obtain the highest viable cell density. VCCs were determined by plating 50 μL of different dilutions of bacterial cell suspensions in Maximum Recovery Diluent (MRD; Oxoid) onto YCFA agar using a Spiral Plater (WASP S00600, Don Whitley, UK). Following 24–48-h incubation, the colonies were counted using a Synbiosis ProtoCOL3 colony counter with software version 1.054 (Synbiosis, UK). Cell pellets were frozen at − 20 °C until further use. For analysis, frozen bacterial pellets were thawed for 5 min at room temperature, tubes were centrifuged at 12000×*g* for 3 min, and any residual supernatant was removed. Pellets were resuspended in 1 mL of pure water (Chromasolv^™^ LC-MS grade, Honeywell, UK). To concentrate or dilute the cell material, the resuspended sample was centrifuged again (12,000×*g*, 3 min) and concentrated or diluted by adding different volumes of pure water (see Electronic Supplementary Material (ESM), Excel file). VCC per mL of the fresh late log cultures was used to calculate a VCC per mL value for each dilution or concentration step. For each dilution or concentration, 24 replicates were measured by MALDI-ToF-MS using 1 μL of bacterial suspension. Results were classified following the standard identification scores provided by the Bruker Biotyper^®^ software as described above. According to this classification, samples, whose ID scores fell into the respective categories, were counted.

### Identification of mixed cultures

Mixed cultures were prepared using the same bacterial strains that were used for the determination of the optimal concentration for ID in pure cultures. Mixtures were prepared by combining resuspended pellets of two different bacterial species (three biological replicates per species). As a standard, a dilution factor of 1 was used. For *E*. *gallinarum*, *M*. *massiliensis*, and *E*. *faecalis*, this factor was increased to 4 after the first measurements showed a poor performance (ID scores < 1.7). The resuspended pellets of the different bacterial species were mixed using different proportions within a range of 95 to 5% (vol/vol) (see ESM, Excel file). By using the VCC per mL of the fresh culture at late logarithmic growth phase, the dilution factor and the volumetric proportion, a mixture ratio (*mixR*) was calculated as follows:

$$ mixR={\log}_{10}\frac{VCC1\times dF1\times v1}{VCC2\times dF2\times v2} $$

*mixR*: mixture ratio
*VCC*1: viable cell count per mL of organism 1
*VCC*2: viable cell count per mL of organism 2
*dF*1: dilution factor of organism 1
*dF*2: dilution factor of organism 2
*v*1: vol.% of organism 1 in the mixture
*v*2: vol.% of organism 2 in the mixture

A total of 1 μL of each mixture was identified using the MALDI-ToF in sextuplicate. The flexAnalysis software (Bruker, Germany) was used to process the spectra. The measurement of a mixed culture returned three values: The first two ID scores (*Sc*1 and *Sc*2) were yielded by the comparison of the measured spectrum with each of the two reference spectra of the bacterial species in the mixture. The third value *Scmix*, the mixed culture algorithm, created an overlaid sum spectrum merging the two single database reference spectra. Whenever *Scmix* was higher than *Sc*1 and *Sc*2, the measured spot was counted as a positively identified mixed culture (*IDmix*). If *Sc*1 or *Sc*2 was higher than *Scmix*, the measured spot was identified as a pure culture and counted as *ID1* for organism 1 or *ID2* for organism 2, respectively. If *Scmix*, *Sc1*, or *Sc2* did not appear among the 50 highest scores, the sample was considered to be not detected and a score of 1 was given. An additional analysis was performed to obtain a global comparison of the different combinations expressed in one number. The global success rate (*Gsr*) of the mixed culture algorithm was calculated by counting all measurements identified as a mixed culture divided by the total number of measured ratios. A high *Gsr* indicated that a mixed culture was identified over a wide range of VCC ratios. A respective calculation was performed for the counts of organism 1 and organism 2 to calculate C(*ID*1) and C(*ID*2). In another step, the range of ratios was constricted. At first, all ratios outside the range of *mixR* − 0.95 and 0.95 (*mixR* ± 0.95 = ratios between 10 and 90%) were excluded. As a second step, all ratios outside the range of *mixR* − 0.48 and 0.48 (*mixR* ± 0.48 = ratios between 25 and 75%) were excluded. The global success rate (*Gsr*) was then calculated without considering ratios outside the respective range.

$$ Scmix> Sc1 and\  Sc2\Rightarrow IDmix={\sum}_{i=1}^61 $$

$$ Sc1> Scmix\ and\  Sc2\Rightarrow ID1={\sum}_{i=1}^61 $$

$$ Sc2> Scmix\ and\  Sc1\Rightarrow ID2={\sum}_{i=1}^61 $$

$$ Gsr=\frac{\mathrm{Number}\ \mathrm{of}\  IDmix}{\mathrm{Total}\ \mathrm{number}\ \mathrm{of}\  mixR\ \mathrm{measured}} $$

*Scmix*: score obtained by applying the mixed culture algorithm
*Sc*1: score obtained for organism 1
*Sc*2: score obtained for organism 2
*IDmix*: number of measured spots with a positive mixed culture ID
*ID*1: number of measured spots with a positive ID of organism 1
*ID*2: number of measured spots with a positive ID of organism 2
*Gsr*: global success rate

## Results

### Incubation time

The highest ID scores of duplicate measurements of three biological replicates per bacterial species are summarized in Table 2. As expected, the ID scores of bacterial colonies decreased over an incubation time of 21 days. Differences in the extent of the ID score decrease as well as the time of ID score decrease were observed between the different bacterial species tested (Table 2). The ID scores for *R*. *hominis*, *Parabacteroides distasonis*, *S*. *aureus*, and *E*. *faecalis* were only slightly lower and identified with a score > 2.0 during the whole period of incubation of 21 days. *B*. *stercoris* and *Bifidobacterium breve* also yielded ID scores close to 2.0 throughout the whole incubation period with some data points falling below that mark but did not show a consistent decrease. ID scores for *Bifidobacterium longum* and *Clostridium sporogenes* decreased below 2.0 from day 11 to day 7, respectively, but never fell below that level. *E*. *coli* also showed decreased ID scores below 2.0 after 13 days, which decreased even further to scores < 1.7 (not reliable identification) after 19 days. In contrast, ID scores for *P*. *aeruginosa*, *S*. *Typhimurium*, *M*. *massiliensis*, and *B*. *subtilis* decreased quickly to values below 2.0 after a few days and fell to < 1.7 after 6 days, 10 days, and 14 days, respectively. Looking at the spectra from different time points revealed the cause for the low ID score, as, e.g. peaks of *M*. *massiliensis* already started to disappear after 3 days, whereas the majority of peaks remained present in the spectra of *R*. *hominis* even after 21 days (see ESM Fig. S1).

**Table 2:**
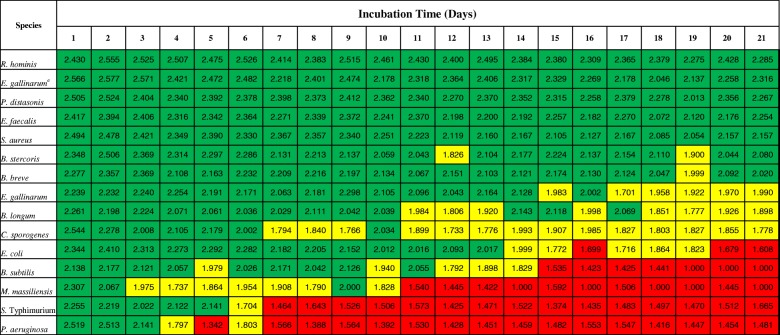
Daily maximum MALDI-ToF ID scores (*n* = 6, duplicate measurements of 3 biological replicates) from bacterial colonies incubated anaerobically at 37 °C for 21 days

^a^Results including custom created MSP for *E*. *gallinarum*

### Identification of single bacterial species from liquid cultures at different cell concentrations

To determine the concentration limits for reliable identification scores at species level (ID scores > 2.0) in liquid media, cell pellets of five Gram-positive (*B*. *breve*, *C*. *sporogenes*, *E*. *faecalis*, *E*. *gallinarum*, *S*. *aureus*) and four Gram-negative (*E*. *coli*, *M*. *massiliensis*, *P*. *distasonis*, *S*. *Typhimurium*) bacterial strains were harvested from liquid cultures at late logarithmic growth and frozen at − 20 °C prior to preparation of different cell concentrations for MALDI-ToF Biotyper^®^ identification. To verify that frozen bacterial cell material resulted in the same MALDI-ToF ID scores as fresh bacterial cell material, fresh and previously frozen cell pellets (− 20 °C, 66 days) from *C*. *sporogenes*, *E*. *faecalis*, and *M*. *massiliensis* were resuspended and measured on the MALDI-ToF. Unlike colonies incubated on agar plates for 21 days, frozen pellets did not show a decrease in maximum ID scores over the frozen storage period of 66 days (ESM Fig. S2).

**Fig. 1:**
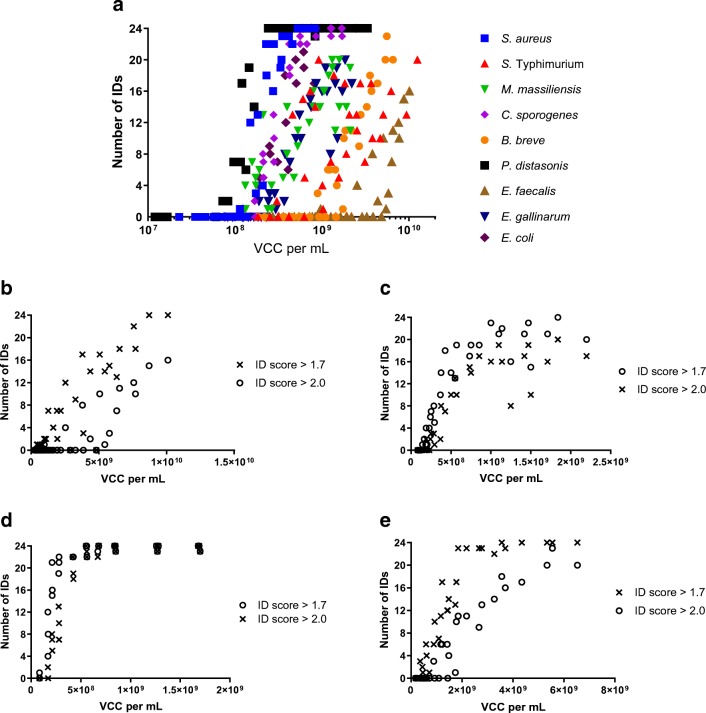
(**a**) Number of Identifications with an ID score > 2.0 (*n *= 24) at different concentrations of VCC per milliliter (in logarithmic scale). Number of Identifications with an ID score > 1.7 and > 2.0 (*n *= 24) of strain (**b**) *E. faecalis,* (**c**) *C. sporogenes,* (**d**) *E. gallinarum,* (**e**) *B. breve*

To determine the minimum bacterial cell concentration with a maximum of successfully identified replicates per sample, previously frozen bacterial cell pellets were thawed and diluted or concentrated to a broad range of VCC per mL (see ESM, Excel file) for MALDI-ToF-MS testing. The dataset of ID scores from these measurements was analyzed by counting the number of samples identified using different quality criteria for the ID score. The number of measurements which achieved ID scores > 1.7 or > 2.0, respectively, improved with an increasing concentration of cells in the bacterial solutions. The data mostly followed a sigmoid distribution (except for *B*. *breve* and *E*. *faecalis*) with a linear increase and a subsequent range of concentrations where the maximum number of identifications (*n* = 24) was achieved (Fig. 1a). For the initial analysis, all samples with an ID score > 1.7 were counted as successfully identified. Subsequently, the same analysis was performed using stricter quality criteria in which only ID scores > 2.0 were counted. The Bruker Biotyper^®^ software reports all samples with an ID score < 1.7 as “not reliable identification” instead of a species ID. To determine the concentration of VCC per mL necessary to avoid a “not reliable identification” result, the linear part of the sigmoid curve was used for linear regression analysis of measurements with ID scores > 1.7 and > 2.0. Linear equations (*y* = slope*x + *y*-intercept) were used to calculate the VCC per mL, at which all 24 measured spots returned an ID score > 1.7 and ID score > 2.0, respectively. For some species, not all 24 measurements were identified successfully even at the highest concentration when the stricter criterium (ID score > 2.0) was applied. In the case of *E*. *faecalis*, the maximum of successful IDs was 16 (Fig. 1b); for *S*. *Typhimurium*, *M*. *massiliensis*, and *E*. *gallinarum*, it was 20 (Fig. 1d, ESM Figs. S3B and S3E). For each organism, the maximum number of measurements which returned an ID was used for calculation (*n* = 24; except for *E*. *faecalis*, *n* = 16; *M*. *massiliensis* and *E*. *gallinarum*, *n* = 20). Application of the Wald–Wolfowitz runs test confirmed no significant deviation from linearity in the data of any species (see ESM, Excel file). The data points of the different species fitted well to their respective lines for both quality criteria. *R*^2^ values ranged between 0.69 and 0.92 (see ESM, Excel file). An exception was *S*. *Typhimurium*, for which the measurements did not fit into a linear distribution (ID score > 1.7, *R*^2^ = 0.54, *p* = 0.21; ID score > 2.0, *R*^2^ = 0.22, *p* = 0.65), and it was therefore excluded from the calculation. For *E*. *faecalis*, the whole range was used to calculate the linear model.

**Fig. 2:**
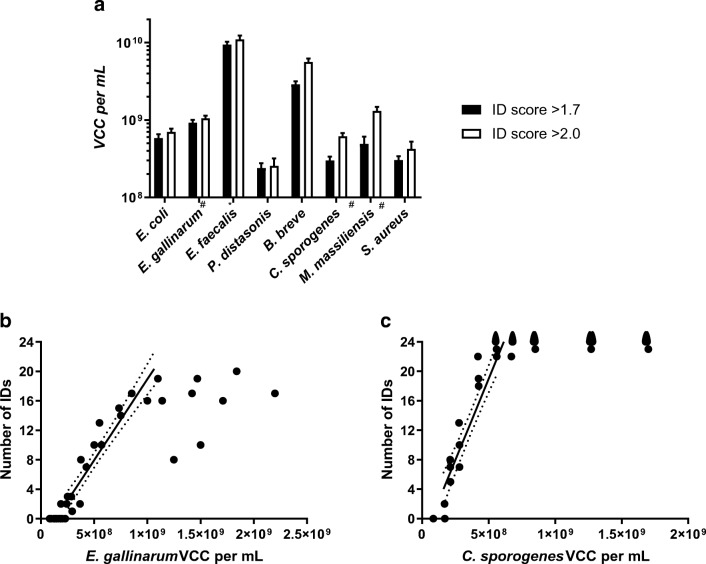
(**a**) Concentration (VCC per milliliter) for a reliable identification (ID score > 1.7 and > 2.0, *n* = 24 successful identifications) of bacteria calculated with a linear regression model. The standard error of the predicted value was determined by a parametric bootstrap estimation (10,000 re-samplings). Linear regression models were calculated using the linear part of the whole range of concentrations measured counting all samples with a reliable ID score (> 1.7 or > 2.0, *n* = 24, # *n* = 20, * *n* = 16). Graph shows *E. gallinarum* (**b**) and *C. sporogenes* (**c**) All concentrations with trend line of the linear range and 95% CI

As expected, the application of lower concentrations of cell material resulted in lower ID scores, with higher VCC per mL required for ID scores > 2.0 compared with > 1.7. The calculated minimum concentration for which 24 measurements would return in an ID score > 1.7 or > 2.0 varied considerably between bacterial species (Fig. 2a). The lowest concentrations (VCC per mL) were required for *P*. *distasonis* (2.40 × 10^8^ for ID scores > 1.7 and 2.55 × 10^8^ for ID scores > 2.0) and *S*. *aureus* (3.04 × 10^8^ for ID scores > 1.7 and 4.24 × 10^8^ for ID scores > 2.0), and the highest ones for *E*. *faecalis* (9.45 × 10^9^ for ID scores > 1.7 and 1.10 × 10^10^ for ID scores > 2.0) and *B*. *breve* (2.89 × 10^9^ for ID scores > 1.7 and 5.62 × 10^9^ for ID scores > 2.0) (Fig. 2a). Species-specific differences in VCC per mL culture required for the maximum number of identifications were observed between quality criteria (ID scores > 1.7/> 2.0). Differences were comparatively low for *E*. *coli* (5.87 × 10^8^/7.02 × 10^8^), *S*. *aureus* (3.04 × 10^8^/4.24 × 10^8^), and *P*. *distasonis* (2.40 × 10^8^/2.55 × 10^8^). High differences were observed for *C*. *sporogenes* (2.99 × 10^8^/6.14 × 10^8^), *B*. *breve* (2.89 × 10^9^/5.62 × 10^9^), and *M*. *massiliensis* (4.94 × 10^8^/1.31 × 10^9^).

### Identification of bacteria in mixed cultures

**Fig. 3:**
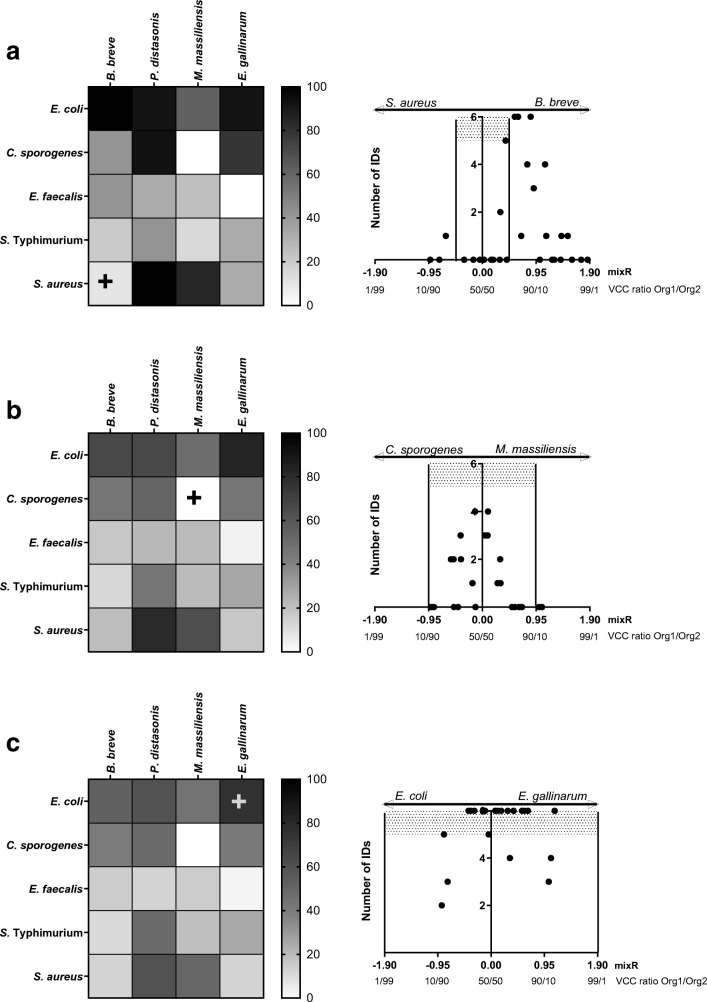
Global success rate (*Gsr*) of combinations containing two different species. Different ratios of two bacteria (*n* = 6) were counted, if positively identified as mixed culture (**a**) within a *mixR* ± 0.48, (**b**) within a *mixR* ± 0.95, (**c**) all measured ratios without constrictions. Example graphs of representative combinations are highlighted by a “+” in the heatmaps and displayed on the left. Grey area displays the range used to calculate *Gsr* values of the respective heatmap, which is defined by the minimum number of mixed culture IDs (> 4 of 6) and the range of *mixR*

Twenty different combinations of mixed cultures containing two different species in different concentrations were measured using the mixed culture algorithm of the Bruker Biotyper^®^ software to determine if the samples were successfully identified as mixed cultures. Failure to identify mixed cultures would result in incorrect identification as a pure culture of one of the two organisms within the culture. For each combination, at least 21 different mixture ratios (see ESM, Excel file) were measured as 6 replicates. If at least 5 replicates were identified as a mixed culture, the identification of a certain *mixR* was considered successful. The global success rates (*Gsr*) were calculated (1) considering all *mixR*, (2) excluding ratios outside a *mixR* value ± 0.95 (ratios between 10 and 90%), and (3) excluding ratios outside a *mixR* ± 0.48 (ratios between 25 and 75%). The *Gsr*s were dependent on the bacterial species in the mixtures and are presented in Fig. 3. The *Gsr*s were relatively low when calculated including all mixture ratios and increased in most cases when extreme ratios were excluded. In the majority of combinations, the highest number of successfully identified mixed cultures was close to a *mixR* value of 0, which corresponds to an equal number of VCC per mL in the mixture for each bacterial species and is reflected by the increase of the average *Gsr* after excluding extreme ratios. For example, the combinations of *B*. *breve* with *E*. *coli* achieved a *Gsr* of 53% by considering all measured *mixR*, which increased to 67% by excluding ratios outside a *mixR* ± 0.95, and to 100% by excluding ratios outside a *mixR* ± 0.48. Similar results of strongly increasing *Gsr* with increasing restriction of ratios show the combinations of *E*. *gallinarum* and *E*. *coli* (76%, 83%, 92%) and *P*. *distasonis* and *S*. *aureus* (60%, 80%, 100%). A much lower *Gsr* increase was observed for the combination of *M*. *massiliensis* and *E*. *faecalis* (15%, 22%, 25%) and for the combination of *B*. *breve* and *S*. *Typhimurium* (14%, 16%, 27%). However, a higher level of constriction for the *Gsr* calculation led to a decrease in the *Gsr* if there was a strong bias towards one organism. This was true for the combinations of *M*. *massiliensis* and *S*. *Typhimurium* with a small increase (from 19 to 22%) after excluding *mixR* outside ± 0.95 but a decrease after excluding *mixR* outside ± 0.48 to a *Gsr* of 15%. ESM Fig. S4D illustrates that a mixture containing equal VCCs of *M*. *massiliensis* and *S*. *Typhimurium* is incorrectly identified as *M*. *massiliensis*. Only after *S*. *Typhimurium* began to outnumber *M*. *massiliensis* was the mixed culture correctly identified. Interestingly, even mixtures containing low concentrations of *M*. *massiliensis* (2.6 × 10^8^ VCC per mL vs. *S. Typhimurium* 2.3 × 10^9^ VCC per mL) were correctly identified despite the big differences in numbers. For *P*. *distasonis* and *S*. *Typhimurium*, a constant decrease in Gsr with further excluding ratios could be detected (48%, 46%, 42%) (Fig. 3). The strong bias towards *P*. *distasonis* can be seen in ESM Fig. S5C.

For all tested *mixR*, *S*. *Typhimurium* was never identified as pure culture, although 2.8 × 10^9^ VCC per mL *S*. *Typhimurium* were mixed with 1.5 × 10^8^*P*. *distasonis* (*mixR* − 1.29) (ESM Fig. S5C). Clear biases towards one species in the mixtures were observed in seven further combinations (ESM Figs. S4–S6). There were two combinations in which none (*M*. *massiliensis* with *C*. *sporogenes*, Fig. 3b) or only one (*E*. *gallinarum* with *E*. *faecalis*, ESM Fig. S5F; *Gsr* values with increasing restriction3%, 4%, 0%) of the measured *mixR* returned more than four positively identified mixed cultures.

### Growth curves and viable cell counts

The growth curves, VCCs of the fresh cultures in late logarithmic growth phase, the calculated values of VCC per mL of each dilution/concentration, and the ID scores of all measured spots for determination of the ideal concentration of VCC per mL for identification of pure cultures as well as the evaluation of the mixed culture algorithm are provided in the ESM Excel file, ESM Fig. S7, and ESM Fig. S8.

## Discussion

The current work clearly demonstrates that the reliable identification by MALDI-ToF-MS over time is highly variable between different bacterial species, as shown by the long-term incubation of colonies from phylogenetically different bacterial species. While some species (*R*. *hominis*, *P*. *distasonis*, *E*. *gallinarum*, *E*. *faecalis*, *S*. *aureus*) continued to be identified with scores > 2.0 throughout the study period of 21 days, others (*M*. *massiliensis*, *P*. *aeruginosa*) showed a dramatic drop of ID scores within a few days. It appears that species resulting in high ID scores over many days possess proteins which are more stable than those of other species. There are only a few reports of using long-term incubated biomaterial, and the incubation times in those studies are much shorter than in the present work. For instance, McElvania Tekippe and co-workers [26] sub-cultured 28 Gram-positive bacteria (including *S*. *aureus* and *E*. *faecalis*) over five consecutive days, which led to a decrease in identification success. In another study, the authors incubated 20 different anaerobic Gram-negative and Gram-positive bacteria for 4 days without showing a decrease in ID scores or a different pattern of Gram-positive and Gram-negative organisms [27]. In the present work, similar results were achieved for most of the species, yielding ID scores ≥ 2.0 after 4 days. For best chances of a reliable identification, colonies should not be older than 2 days, as even the most sensitive species could be identified with a score > 2.0 after this time.

ID scores are calculated by matching measured spectra with reference spectra (MSPs). The manufacturer database contains more than one MSP for many species, due to factors such as intra-species variability and different culturing conditions which can impact ID scores. In the present work, a higher number of MSPs for a particular bacterial species in the manufacturer’s database (BDAL) did not have a positive effect on ID scores. The creation of a custom MSP in this work resulted in higher ID scores, which confirms that custom MSPs created from a specific bacterial strain grown under specific conditions improve identification performance. This may be due to the exclusion of possible intra-species variation including different growth medium requirements among the different strains. Here again, no link between the cell wall structure (Gram-positive vs. Gram-negative) of bacterial species was observed; rather, our data showed high inter-genera and inter-species variability in general.

Apart from the age of the fresh biomaterial, successful identification and ID scores have been shown to be influenced by the concentration of biomaterial applied for MALDI-ToF identification of bacteria ([12, 15, 28]). Increasing concentrations of biomaterial result in increased number of successful identifications and higher ID scores in repeated measurements. Detection limits reported for measurements of *E*. *coli* differed, when dilutions were measured on an Autoflex III [12], an Ultraflex MALDI-ToF/ToF [15], or a Bruker Microflex LT mass spectrometer [13] (present study). In contrast to other studies, we determined the minimum bacterial concentration required for the maximum success of identification of a large variety of phylogenetically different bacteria as a single value by applying a linear regression model.

If pre-adjusted quality criteria are not met (such as signal-to-noise threshold, minimum intensity threshold), then, at certain levels, low amount of biomass results in flatline spectra. Throughout all experiments, we observed that ID scores of technical replicates tended to vary occasionally. Different factors, such as the optimal amount of biomass, the process of drying the sample, and the matrix on the target spot, produce a certain amount of variability. The subsequent formation of crystals can lead to an unequal distribution of areas yielding high peaks and ones without any detectable peaks at all [29]. Flatline spectra were not excluded from the present dataset, reflecting the behavior of bacterial samples in day-to-day routine measurements. To minimize the effect of random flatline spectra, a number of replicates were measured in each experiment.

The visual inspection of colony morphologies on agar plates can give a first hint of the presence of different bacterial species. As this is not possible in liquid cultures, we evaluated the sensitivity and accuracy of MALDI-ToF identification of mixed cultures from bacterial solutions. Our data show that the success of mixed culture identification is highly variable and depends on the combination of species and their respective concentration. These results are in line with previous studies, where different volumetric ratios of various mixed bacterial solutions were tested [17, 19]. Defining mixtures as volumetric ratios, however, can be a problem, if the species combined show a high difference in concentration. The study of Mahe and colleagues reported that by using volumetric mixtures of two pure liquid cultures, one combination diverged clearly from their predicted model, although both pure cultures had equal values of optical density [17]. The same differences were reported by Zhang and colleagues in a six species mixture [18]. In another recent approach, a set of bacteria were mixed based on OD_600_ values and a cell count was conducted using selective media [22]. Although selective media can prohibit non-target microorganisms from growth, they usually have a certain range of recovery rates concerning target organisms. Hence, the VCC of a bi-microbial mixture using a selective agar does not necessarily resemble a true VCC as performed in the present study. McFarland standards or OD_600_ values are not appropriate for exact determinations of concentrations in bacterial cultures, as the turbidity of a culture does not correspond directly to cell counts in bacterial solutions of different species (growth curve and VCC data of this study provided in the ESM Excel file, ESM Fig. S7, and ESM Fig. S8). Hence, for the analysis of mixed cultures, in this study, the VCCs of each bacterial species were taken into account for the calculation of the mixture ratios (*mixR*).

Our results show very variable *Gsr*s depending on the species combinations measured for the mixed cultures. Low *Gsr*s may be attributed to several different factors. The fact that *E*. *faecalis* showed comparatively low *Gsr*s in all combinations, although it was mixed using a fourfold concentrate of the original culture, could be related to its poor performance as a pure culture, whereas the opposite was the case for *E*. *coli*. Similar findings are described by another study [19], where a high volumetric ratio of 9:1 (*E*. *faecalis*:*E*. *coli*) was necessary for mixed culture identification, whereas the ratio 1:2 samples were incorrectly identified as *E*. *coli* pure cultures. In our work, this kind of bias towards one of the microorganisms in mixtures was observed for nine combinations. This effect has been described in another study as a “dominance” of one organism in mixture over the second one [9]. The phylogenetic distance of the bacterial species in the mixture also seems important for mixed culture identification. Mahe and colleagues [17] tested different combinations of mixtures, which involved samples of two microorganisms of the same genus. This approach showed that the success of mixed culture detection decreases with phylogenetic proximity. In the present work, similar results were found for the combination of *E*. *gallinarum* and *E*. *faecalis*. The theoretical sum spectrum of two pure culture MSPs, which is applied by the mixed culture detection algorithm, relies on peaks with different *m*/*z* (mass-to-charge ratio). Spectra of microorganisms of the same genus partly share the same peaks; as shown for *E*. *gallinarum* and *E*. *faecalis* in this study, it is therefore more difficult to identify mixtures of closely related organisms, as reflected by the very low *Gsr* for closely related organisms. Therefore, it would be very difficult to identify a contaminant that is phylogenetically close to the microorganism of interest.

This work shows that successful identification of bacterial species using MALDI-ToF-MS varies depending on the incubation time of colonies and the cell concentrations of pure bacterial cultures and of bi-microbial mixtures. Although most tested microorganisms can be identified successfully after a long incubation time, colonies should be measured within 2 days after colonies have grown to reduce negative effects on ID performance. Freezing does not affect the obtained ID score; hence, in the case of high sample numbers, bacterial pellets can be frozen and measured later. Furthermore, for repeated measurements of the same known organism at given growth conditions, a custom MSP will improve identification performance.

The present study shows that bacterial suspensions below the concentration 10^8^ VCC per mL are not concentrated enough to be identified on the Bruker Microflex MALDI-ToF-MS, and for some bacterial species, this need to be considerably higher. This finding is important for biotechnology applications where bacterial biomass is produced and indicates that most bacterial suspensions will need to be concentrated before identification. Furthermore, the strong variations between different species limit the use of MALDI-ToF-MS for detecting mixed cultures (e.g., contaminations) in bacterial biomass produced from liquid cultures. However, it can be a valuable pre-screen tool for quick and early detection before proceeding further with more time-consuming methods such as traditional cultivation or molecular biology including sequencing.

## Electronic supplementary material

ESM 1 (PDF 342 kb)

ESM 2 (XLSX 335 kb)

## Abbreviations

BTS: Bacterial Test Standard
*Gsr*: Global success rate
HCCA: α-Cyano-4-hydroxybenzoic acid
MALDI-ToF-MS: Matrix-assisted laser desorption ionization-time-of-flight mass spectrometry
MRD: Maximum Recovery Diluent
MSPs: Main spectrum profiles
OD_600_: Optical density at 600 nm
TFA: Trifluoroacetic acid
VCC: Viable cell count
WASP: Whitley Anaerobic Spiral Plater
YCFA: Yeast extract casitone fatty acid medium
