# Supplementary material for: Identification performance of MALDI-ToF-MS upon mono- and bi-microbial cultures is cell number and culture proportion dependent
Source: Anal Bioanal Chem. 2019 Sep 5;411(26):7027–38. doi: 10.1007/s00216-019-02080-x (PMC6834929; doi:10.1007/s00216-019-02080-x)
Supplement: Supplementary file 1 — (PDF 342 kb) [file 216_2019_2080_MOESM1_ESM.pdf]

**Analytical and Bioanalytical Chemistry**

**Electronic Supplementary Material**

# Identification performance of MALDI-ToF-MS upon mono- and bi-microbial cultures is cell number and culture proportion dependent

Christoph Mörtelmaier, Suchita Panda, Iain Robertson, Mareike Krell,
Marilena Christodoulou, Nicole Reichardt, Imke Mulder

Additional file available under 10.1007/s00216-019-02080-x.

A

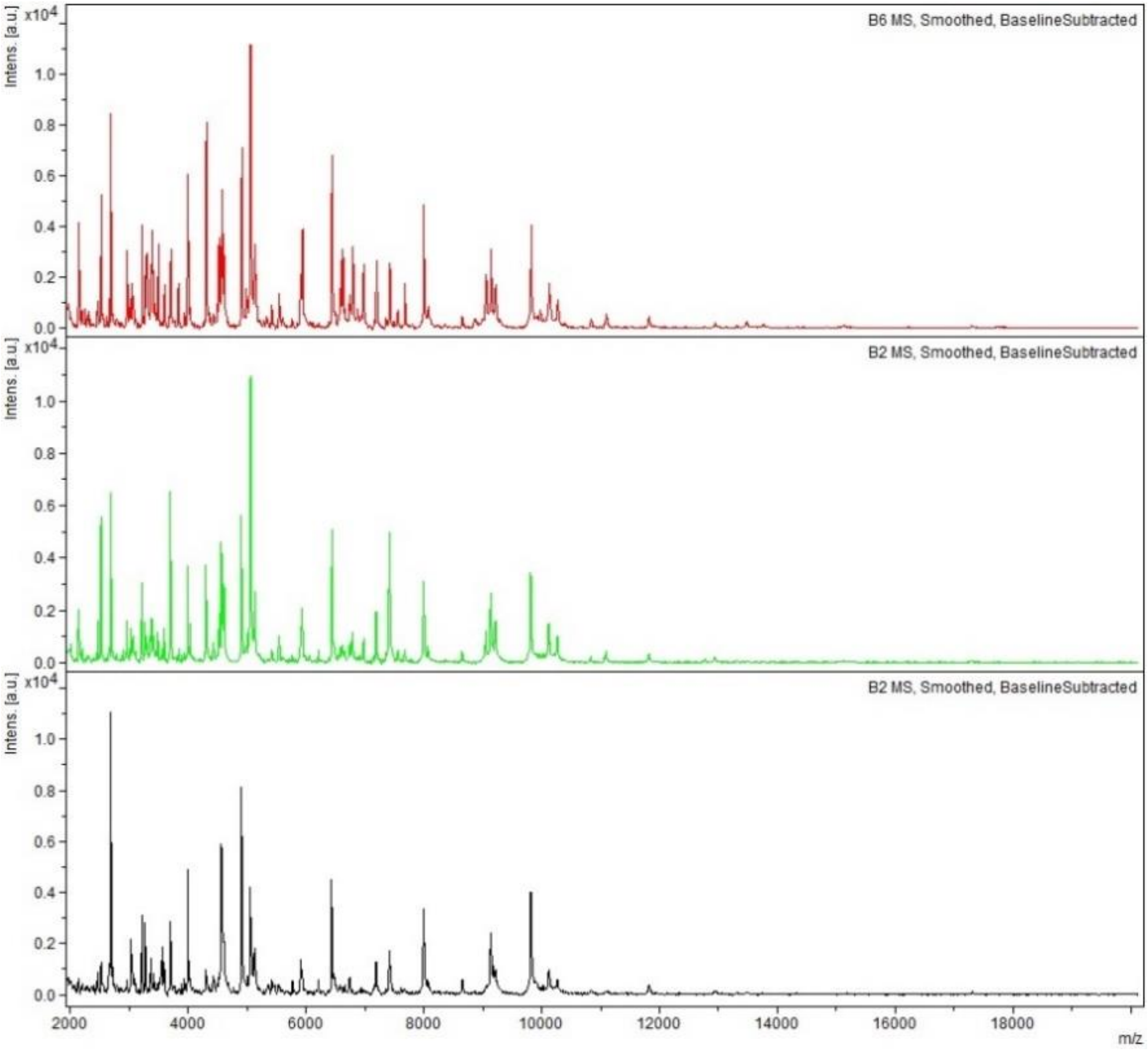
B6 MS, Smoothed, BaselineSubtracted
B2 MS, Smoothed, BaselineSubtracted
B2 MS, Smoothed, BaselineSubtracted
Intens. [a.u.]
x10^4
1.0
0.8
0.6
0.4
0.2
0.0
2000
4000
6000
8000
10000
12000
14000
16000
18000
m/z

B

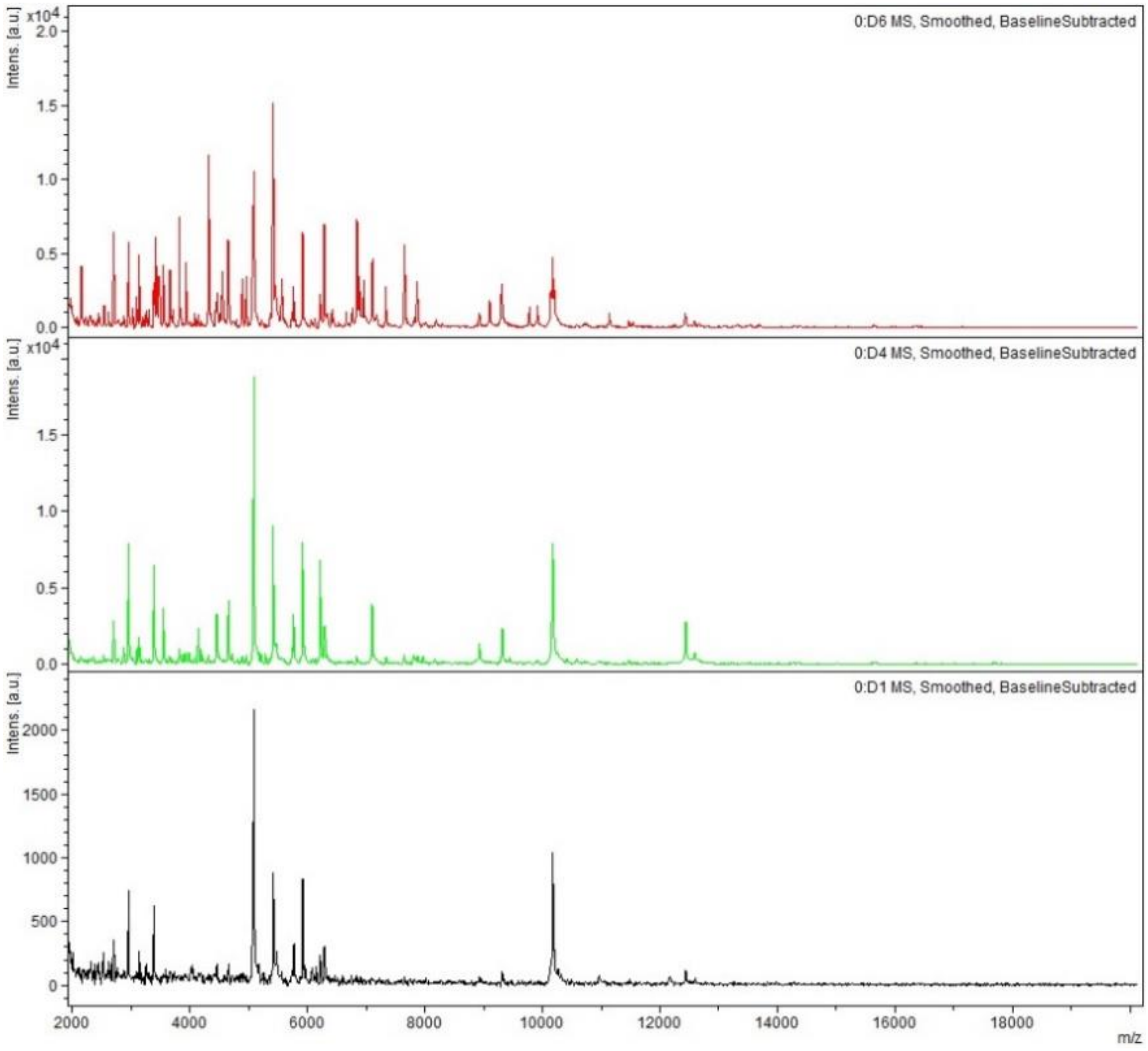


**Fig. S1** Example spectrum (smoothed and baseline subtracted) of colonies measured at different timepoint of incubation on an agar plate A: *R. hominis* after 1 (red, ID score 2.430), 3 (green, ID score: 2.461) and 21 (black, ID score: 2.228) day of incubation. B: *M. massiliensis* after 1 (red, ID score: 2.307), 3 (green, ID score: 1.975) and 5 (black, use of a smaller y axis scale range for better visibility, ID score: 1.864) days of incubation

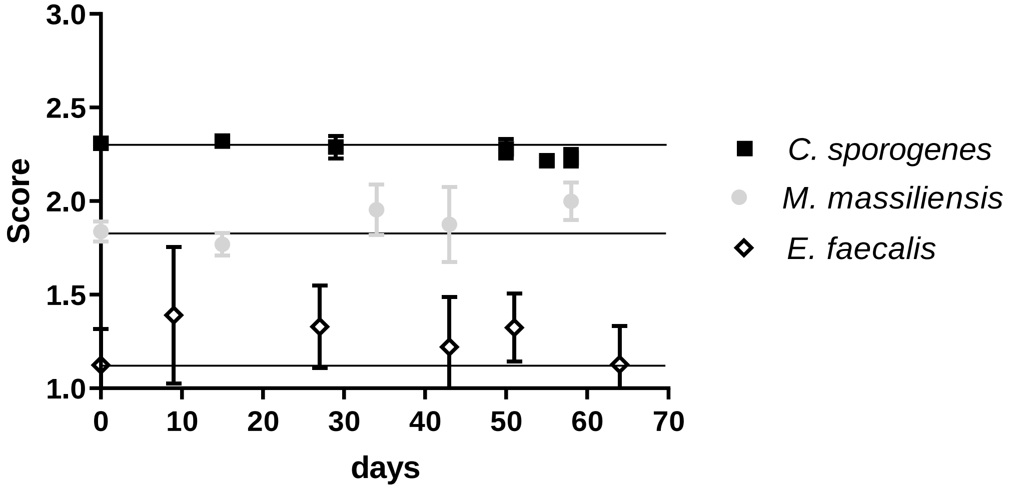


**Fig. S2** Maximum Identification scores (n=6) of *C. sporogenes* (1.68 x $10^9$ VCC per mL), *M. massiliensis* (4.75 x $10^8$ VCC per mL) and *E. faecalis* (1.93 x $10^9$ VCC per mL). Comparison of measurements of fresh pellets (time = 0 days) versus measurements of frozen pellets stored for different amount of time. Horizontal lines show the score level at day 0

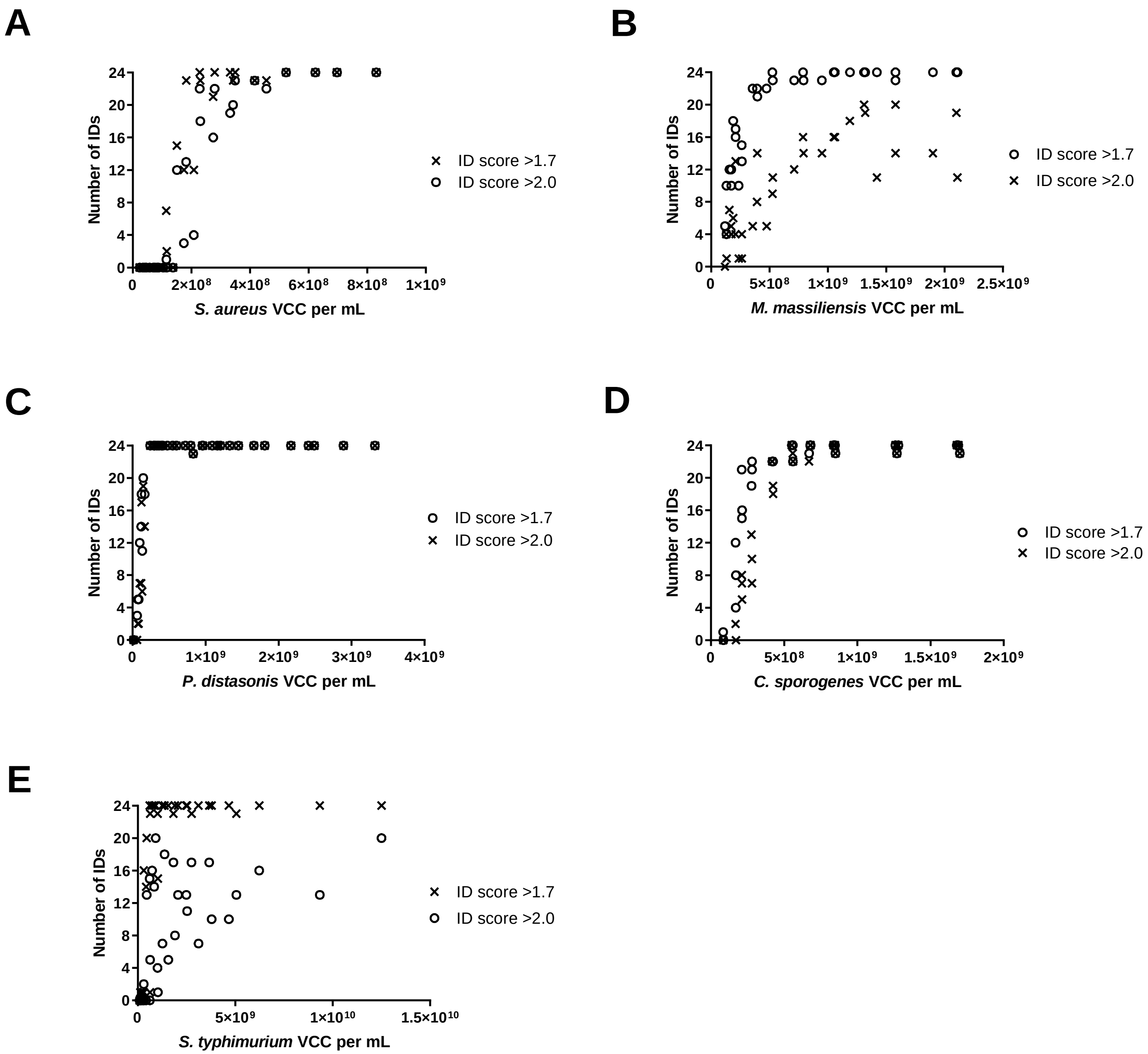


**Fig. S3** Number of Identifications with an ID score >1.7 and >2.0 (n=24) of strain (A) *S. aureus*, (B) *M. massiliensis*, (C) *P. distasonis*, (D) *C. sporogenes*, (E) *S.* Typhimurium.

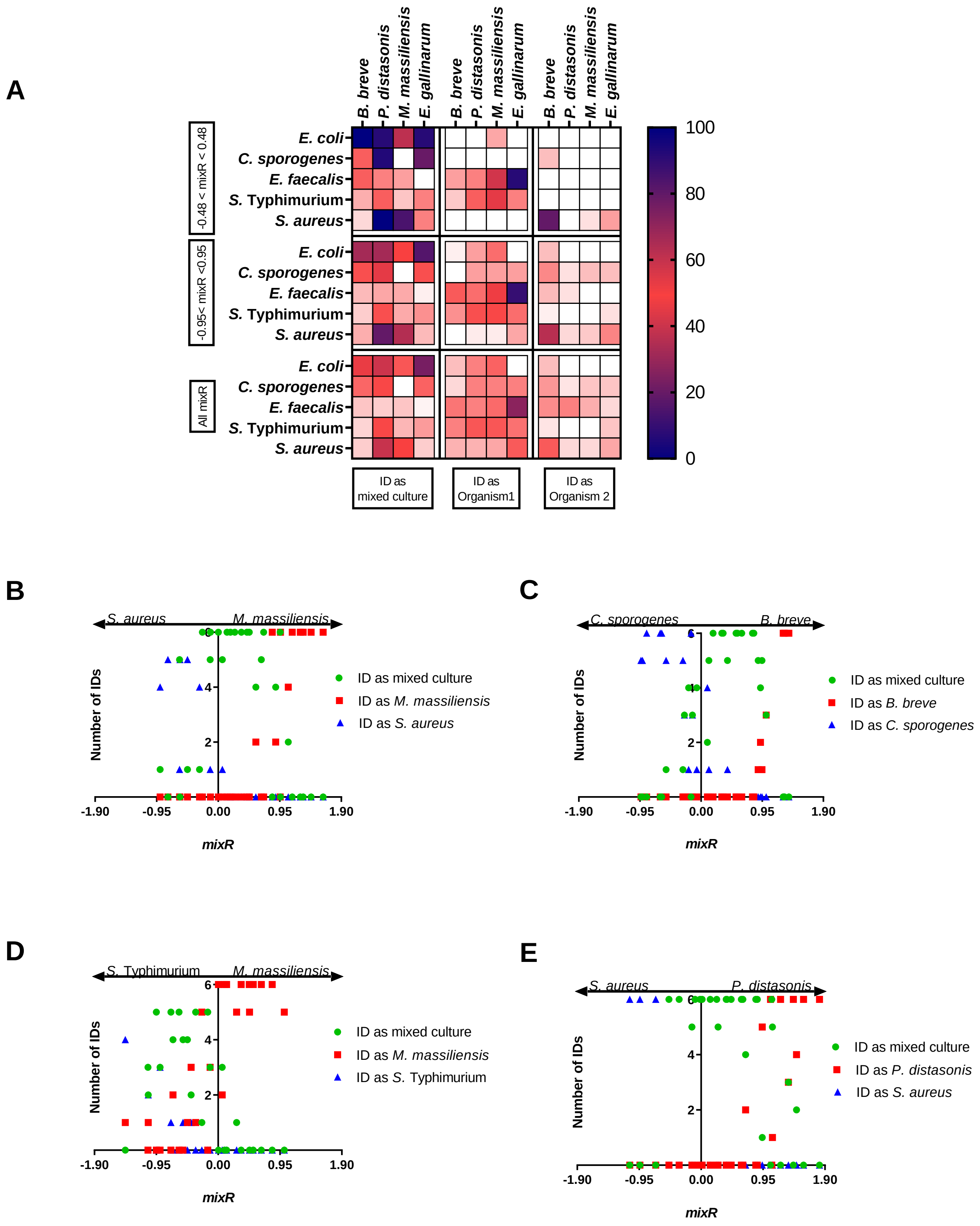


**Fig. S4** (A) Global success rate (*Gsr*) of combinations containing two different species. Different ratios of two bacteria (n=6) were counted; if positively identified as mixed culture (first column), as organism 1 (vertical species names, second column) or as organism 2 (horizontal species names, third column) in more than 4 of 6 replicates in

relation to the total measured ratios. First row: all ratios within a *mixR* ±0.48. Second row: all ratios within a *mixR* ±0.95. Third row: all measured ratios without constrictions. (B) Example graph showing a combination of 2 organisms without inclination and a high *Gsr*. Graph displays the number of mixed culture IDs (n=6) for the combination of *S. aureus* and *M. massiliensis*. (C) Example graph showing a combination of 2 organisms with an inclination towards *C. sporogenes* and a high *Gsr*. Graph displays the number of mixed culture IDs (n=6) for the combination of *C. sporogenes* and *B. breve*. (D) Example graph showing a combination of 2 organisms with an inclination towards *M. massiliensis*, a low *Gsr* and highly inconsistent values. Graph displays the number of mixed culture IDs (n=6) for the combination of *M. massiliensis* and *S.* Typhimurium. (E) Example graph showing a combination of 2 organisms without inclination and a high *Gsr*. Graph displays the number of mixed culture IDs (n=6) for the combination of *S. aureus* and *P. distasonis*

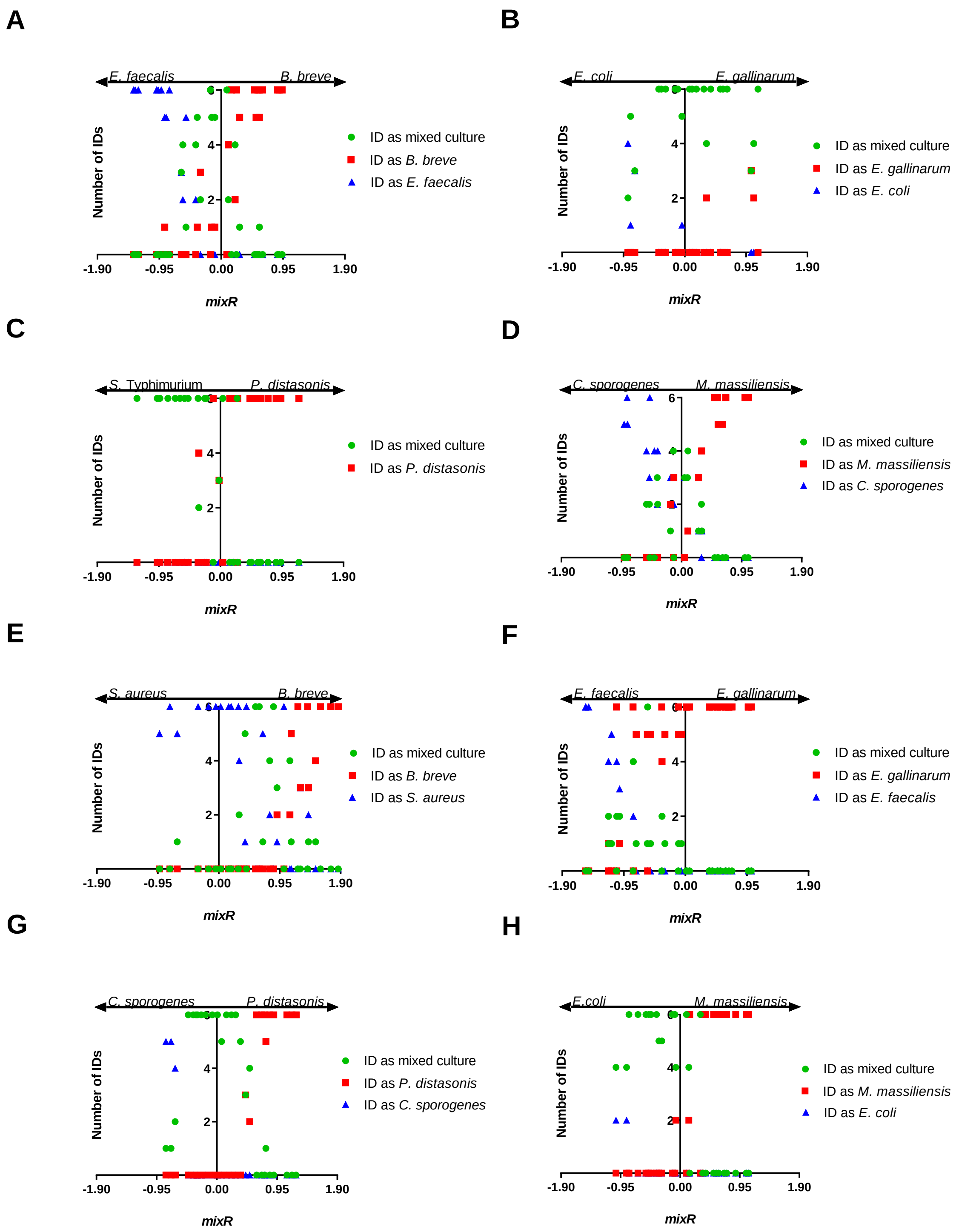


**Fig. S5** (A) Example graph showing a combination of 2 organisms without inclination and a low *Gsr*. Graph displays the number of mixed culture IDs (n=6) for the combination of *E. faecalis* and *B. breve*. (B) Example graph showing a combination of 2 organisms without inclination and a very high *Gsr*. Graph displays the number of mixed culture IDs (n=6) for the combination of *E. gallinarum* and *E. coli*. (C) Example graph showing a combination of 2 organisms with an inclination towards *P. distasonis* and a high *Gsr*. Graph displays the number of mixed culture IDs (n=6) for the combination of *P. distasonis* and *S.* Typhimurium. (D) Example graph showing a combination of 2

organisms without inclination and a *Gsr* of 0. Graph displays the number of mixed culture IDs (n=6) for the combination of *M. massiliensis* and *C. sporogenes*. (E) Example graph showing a combination of 2 organisms with an inclination towards *S. aureus*, a low *Gsr* and highly inconsistent values. Graph displays the number of mixed culture IDs (n=6) for the combination of *S. aureus* and *B. breve*. (F) Example graph showing a combination of 2 organisms with an inclination towards *E. gallinarum* and a very low *Gsr*. Graph displays the number of mixed culture IDs (n=6) for the combination of *E. gallinarum* and *E. faecalis*. (G) Example graph showing a combination of 2 organisms without inclination and a high *Gsr*. Graph displays the number of mixed culture IDs (n=6) for the combination of *C. sporogenes* and *P. distasonis*. (H) Example graph showing a combination of 2 organisms with an inclination towards *M. massiliensis* and a high *Gsr*. Graph displays the number of mixed culture IDs (n=6) for the combination of *E. coli* and *M. massiliensis*

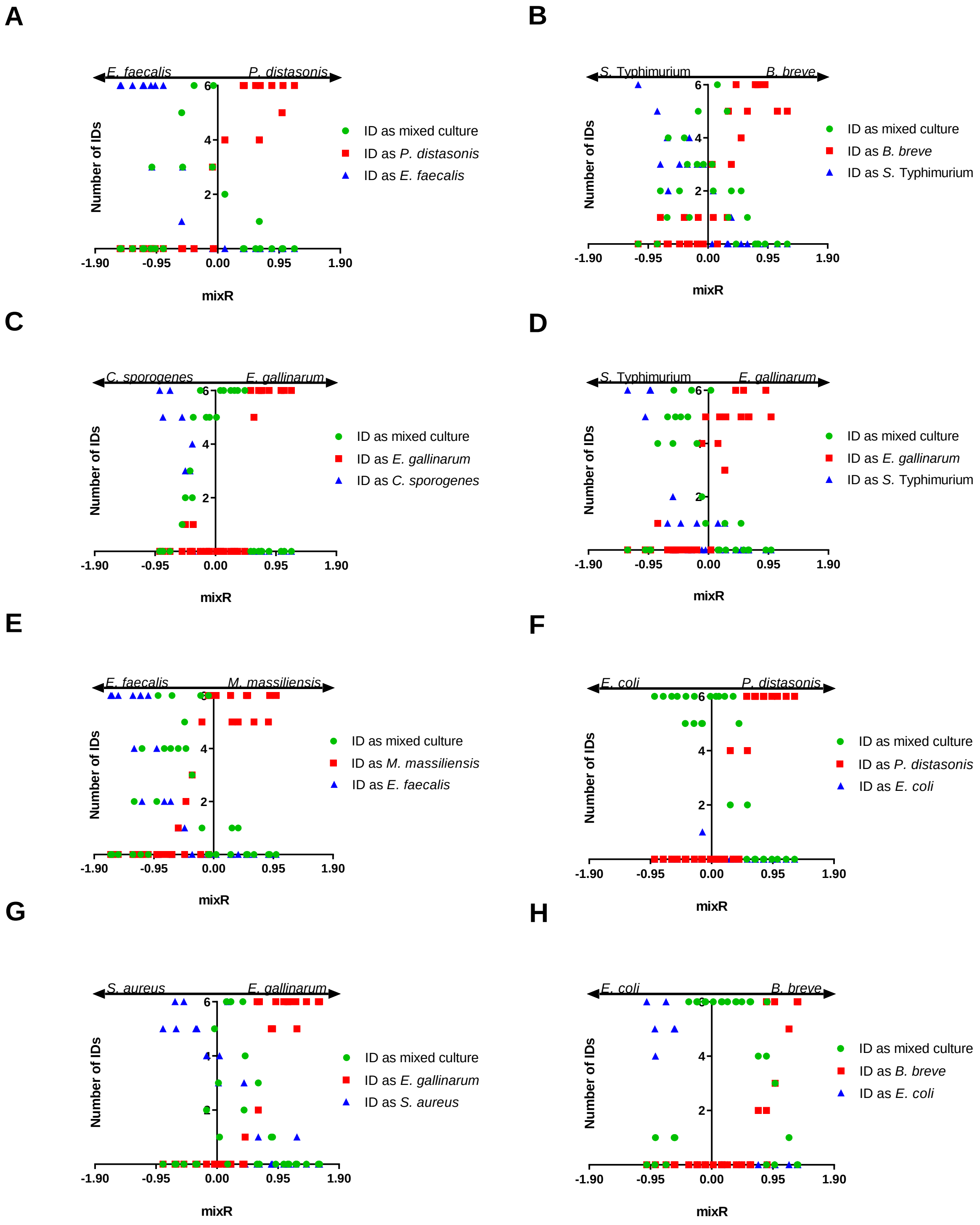


**Fig. S6** (A) Example graph showing a combination of two organisms without inclination and a low *Gsr*. Graph displays the number of mixed culture IDs (n=6) for the combination of *P. distasonis* and *E. faecalis*. (B) Example graph showing a combination of two organisms without inclination and a low *Gsr*. Graph displays the number of mixed culture IDs (n=6) for the combination of of *B. breve* and *S.* Typhimurium. (C) Example graph showing a combination of 2 organisms without inclination and a high *Gsr*. Graph displays the number of mixed culture IDs (n=6) for the combination of *E. gallinarum* and *C. sporogenes*. (D) Example graph showing a combination of 2

organisms with an inclination towards *E. gallinarum* and a low *Gsr*. Graph displays the number of mixed culture IDs (n=6) for the combination of *E. gallinarum* and *S.* Typhimurium. (E) Example graph showing a combination of 2 organisms with an inclination towards *M. massiliensis* and a low *Gsr*. Graph displays the number of mixed culture IDs (n=6) for the combination of *M. massiliensis* and *E. faecalis*. (F) Example graph showing a combination of 2 organisms with an inclination towards *P. distasonis* and a very high *Gsr*. Graph displays the number of mixed culture IDs (n=6) for the combination of *P. distasonis* and *E. coli* number of mixed culture IDs (n=6). (G) Example graph showing a combination of 2 organisms without inclination and a low *Gsr*. Graph displays the number of mixed culture IDs (n=6) for the combination of *E. gallinarum* and *S. aureus*. (H) Example graph showing a combination of 2 organisms without inclination and a high *Gsr*. Graph displays the number of mixed culture IDs (n=6) for the combination of *B. breve* and *E. coli*

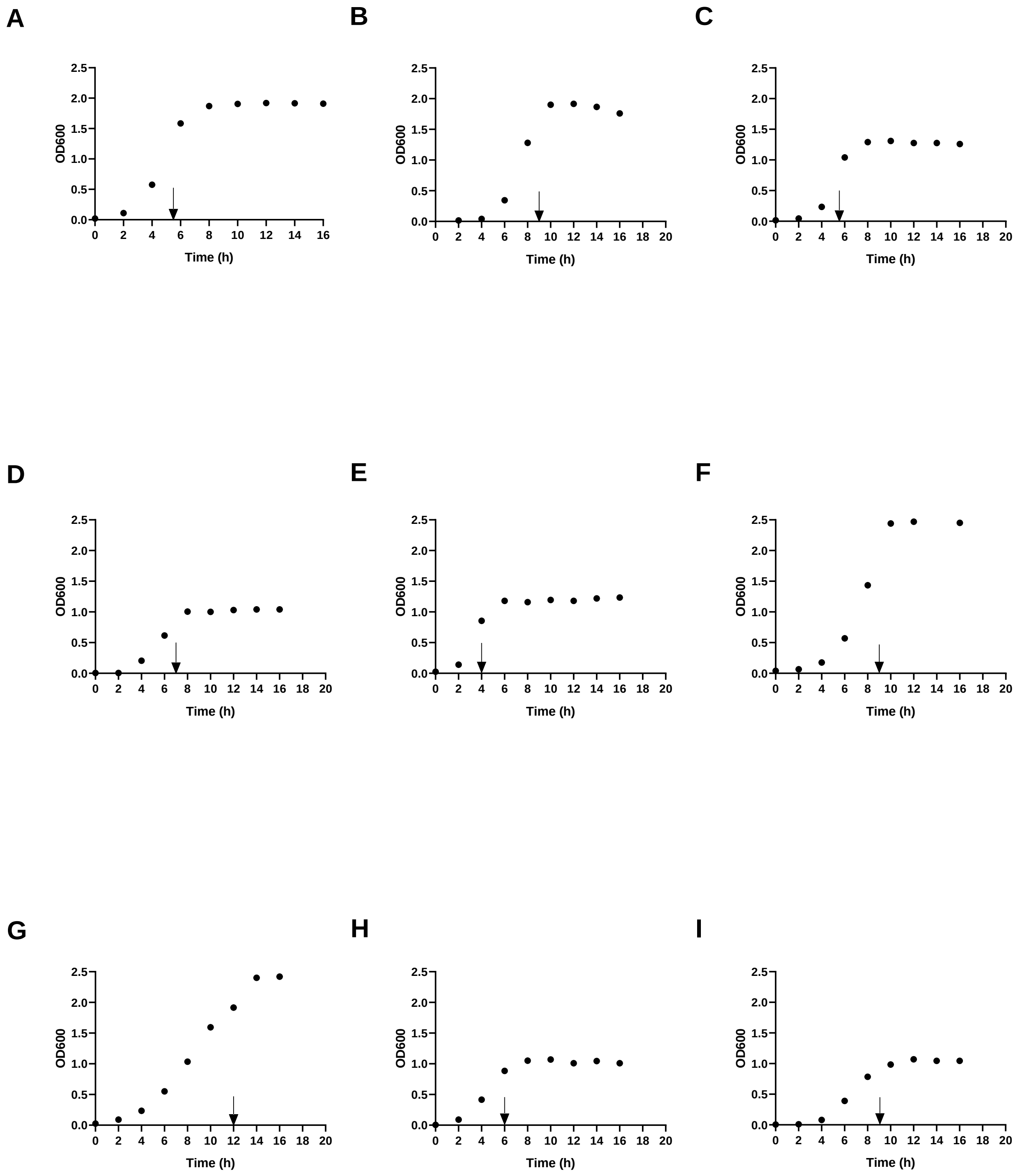


**Fig. S7** Growth curves of 9 different bacteria. Arrow positions indicates the timepoint of sampling for VCC and biomass harvesting. A: *B. breve* sampling at 5.5 h ,B: *C. sporogenes* sampling at 9 h, C: *E. gallinarum,* D: *E. faecalis* sampling at 7 h; E: *E. coli* sampling at 4 h, F: *M. massiliensis* sampling at 9 h, G: *P. distasonis* sampling at 12 h, H: *S.* Typhimurium sampling at 6 h, I: *S. aureus* sampling at 9 h

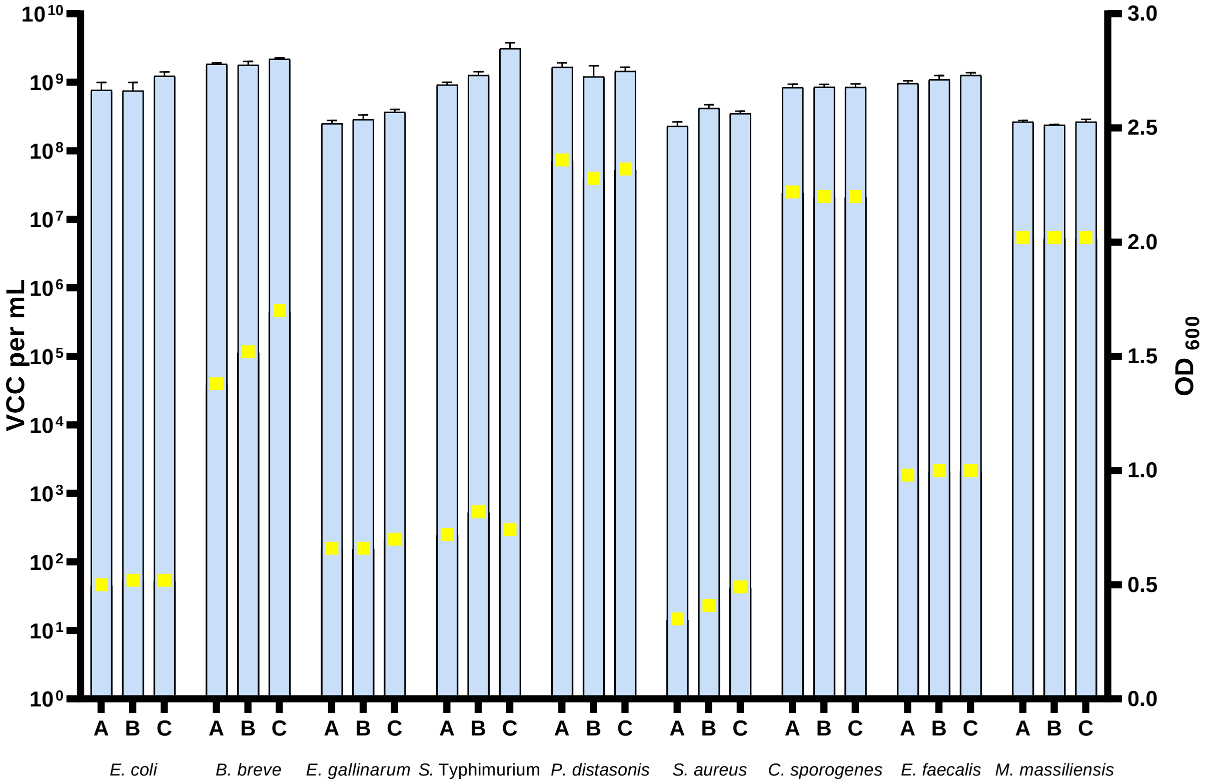


**Fig. S8** Average viable cell count (VCC) and optical density ($OD_{600}$) of bacterial cultures harvested at late logarithmic growth phase
